# Leaf Morphogenesis: Insights From the Moss *Physcomitrium patens*

**DOI:** 10.3389/fpls.2021.736212

**Published:** 2021-09-23

**Authors:** Wenye Lin, Ying Wang, Yoan Coudert, Daniel Kierzkowski

**Affiliations:** ^1^IRBV, Department of Biological Sciences, University of Montréal, Montréal, Montréal, QC, Canada; ^2^College of Life Sciences, University of Chinese Academy of Sciences, Beijing, China; ^3^Laboratoire Reproduction et Développement des Plantes, Ecole Normale Supérieure de Lyon, CNRS, INRA, Université Claude Bernard Lyon 1, INRIA, Lyon, France

**Keywords:** *Physcomitrum patens*, *Physcomitrella patens*, leaf, heteroblasty, bryophytes, development, organogenesis, cellular dynamics

## Abstract

Specialized photosynthetic organs have appeared several times independently during the evolution of land plants. Phyllids, the leaf-like organs of bryophytes such as mosses or leafy liverworts, display a simple morphology, with a small number of cells and cell types and lack typical vascular tissue which contrasts greatly with flowering plants. Despite this, the leaf structures of these two plant types share many morphological characteristics. In this review, we summarize the current understanding of leaf morphogenesis in the model moss *Physcomitrium patens*, focusing on the underlying cellular patterns and molecular regulatory mechanisms. We discuss this knowledge in an evolutionary context and identify parallels between moss and flowering plant leaf development. Finally, we propose potential research directions that may help to answer fundamental questions in plant development using moss leaves as a model system.

## Introduction

Leaves are photosynthetic organs with mainly determinate growth that evolved several times independently during land plant diversification (Tomescu, 2009; Nelissen et al., 2016). These organs can be large with a complex structure as some flowering plant leaves, or small and anatomically simple as bryophyte phyllids (hereafter called leaves). However, they all display a predominantly flat shape as an adaptation to optimize light capture. The genetic basis of leaf development has been extensively studied in flowering plants (Bar and Ori, 2015; Du et al., 2018; Maugarny-Calès and Laufs, 2018). However, how genetic commands are coordinated between cells and translated into supracellular level organization, and the final leaf shape remains largely elusive. This is due to the complex, multilayer structure of flowering plant leaves with interweaving interactions between cells and tissues (Malinowski, 2013).

**FIGURE 1:**
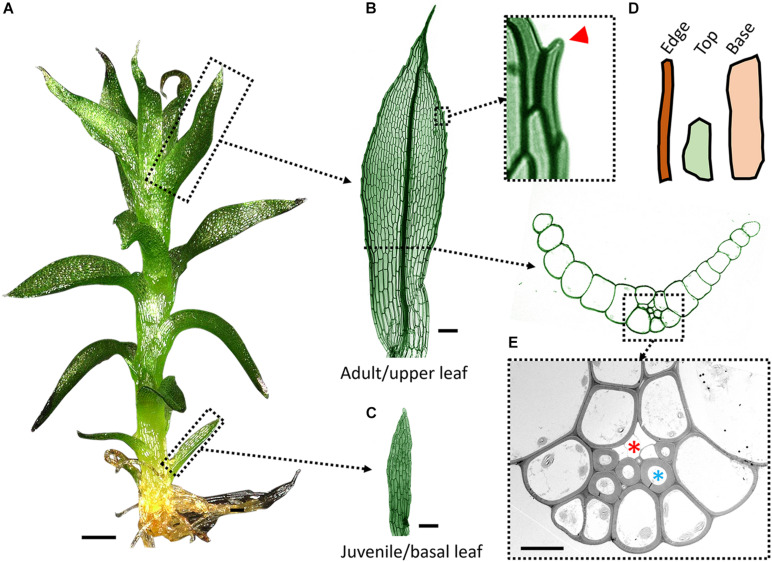
Leaf anatomy in *P. patens*. **(A)** A leafy shoot (or gametophore) with juvenile and adult leaves from the base to the top. **(B)** Adult (upper) leaf has a multicellular midrib and lanceolate shape. The leaf margin is magnified to show marginal serrations (red arrowhead) formed by cell tip outgrowths. **(C)** First juvenile leaf is composed of a single cell layer and has a rectangular shape. **(D)** Three cell shapes are commonly identified in adult leaves: long and narrow cells on the edge; smaller and more isodiametric cells close to the tip (top); long and broad cells near the base (based on Dennis et al., 2019). **(E)** TEM cross-section image of a midrib cell bundle with thick-walled stereids and thin-walled hydroids (marked with blue and red asterisk respectively). Scale bars: 200 μm in **(A)**; 50 μm in **(B,C)**; and 10 μm in **(E)**.

The development of upright bryophyte gametophores, or leafy shoots, has contributed to the colonization of new environments by plants and helped mosses to thrive on land for hundreds of million years (Mitchell et al., 2021). Moss leaves are lateral appendages attached to the stem of gametophores. In the model species *P. patens* (*Physcomitrum patens*, formerly known as *Physcomitrella patens*), leaves are small and composed of cells arranged principally in a single layer (Courtice and Cove, 1983; Figures 1A–C). As the gametophore grows, leaves start to develop a midrib (a bundle of specialized conducting cells) and marginal serrations (Sakakibara et al., 2003; Barker and Ashton, 2013; Dennis et al., 2019; Figure 1E). At first glance, the lanceolate-shaped leaves of *P. patens* mirror the dominant leaf shape of flowering plants, but they have a much simpler structure and smaller size, and can be more easily imaged, which makes them an ideal system for studying leaf development. However, our understanding of moss leaf organogenesis is still limited. Here, we review current knowledge on *P. patens* leaf organogenesis, focusing on the cellular dynamics and molecular factors underlying leaf development.

## Leaf Initiation

**FIGURE 2:**
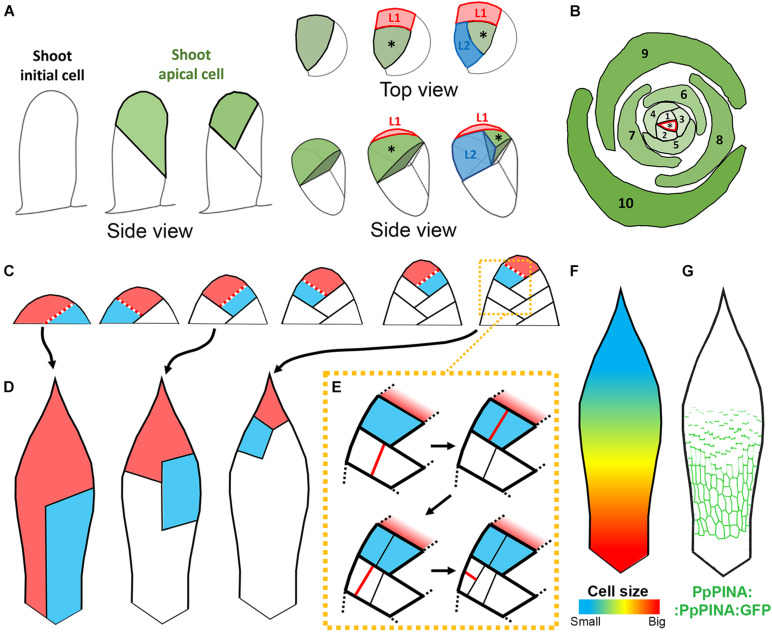
Leaf growth in *P. patens.* **(A)** Schematic representation of the transition from 2D to 3D growth during leafy gametophore initiation. Gametophore shoot initial cell divides obliquely to generate the shoot apical cell (in green). Second and third divisions of the shoot initial cell are also oblique and generate a hair cell (in white) and the first leaf initial (L1, in red) consecutively. From this stage, the shoot apical initial cell becomes tetrahedral and generates subsequent leaf initials. **(B)** A single apical cell (marked with asterisk) produces leaves in a spiral phyllotactic pattern with leaf sequences indicated in consecutive numbers. **(C)** Schematic representation of the early development of the juvenile leaf. The leaf apical cell is shown in red and the cell recently cleaved from the apical cell is shown in blue. Red dotted lines indicate recent cell division (based on Harrison et al., 2009). **(D)** Schematic representation of clonal sectors arising from single cells shown in **(C)** at early stages of leaf development. **(E)** Schematic representation of cell division patterns within the segments generated by the leaf apical cell. Red lines represent new walls (based on Harrison et al., 2009). **(F)** Schematic representation of the distribution of cell sizes in the adult leaf. Bigger cells are located in the distal region of the leaf, smaller cells in proximal region (based on Dennis et al., 2019). **(G)** Schematic representation of the PpPINA protein localization (in green) in the adult leaf (Viaene et al., 2014).

In contrast to flowering plants where lateral organs are generated at the multicellular shoot apical meristem (Kuhlemeier, 2017), leaves in bryophytes are derived from a single shoot apical cell (Gifford, 1983; Harrison et al., 2009). This shoot apical cell is itself generated from a single shoot initial cell. Specification of the shoot initial cell requires both cytokinin and auxin (Ashton et al., 1978; Cove et al., 2006; Bennett et al., 2014). Factors including *DEFECTIVE KERNEL 1* (*DEK1*), *NO GAMETOPHORES 1* and *2* (*NOG1* and *2*) *RECEPTOR-LIKE PROTEIN KINASE 2* (*RPK2*), and *CLAVATA* (*CLV*) function through APETALA2-type (AP2-type) transcription factors to control the frequency of shoot initial cells (Aoyama et al., 2012; Perroud et al., 2014; Moody et al., 2018, 2021; Whitewoods et al., 2018; Demko et al., 2021; Nemec Venza et al., 2021). In *P. patens*, a shoot initial cell undergoes several rounds of stereotypic, oblique cell divisions that lead to the formation of a tetrahedral shoot apical cell, marking the transition from a so-called 2D to 3D growth mode (Figure 2A; Harrison et al., 2009). These divisions are also regulated by *DEK1*, *CLV*, *NOG1*, and *NOG2* genes (Perroud et al., 2014; Moody et al., 2018, 2021; Whitewoods et al., 2018; Nemec Venza et al., 2021), and precisely fulfilled by mitotic spindle orientation regulators, including microtubule-associated protein TARGETING FACTOR FOR Xklp2 and SABRE (Kosetsu et al., 2017; Kozgunova et al., 2020; Cheng and Bezanilla, 2021). Additionally, SOSEKI proteins might also be involved in apical cell identity specification and division (van Dop et al., 2020).

The self-renewing activity of the shoot apical cell gives the gametophytic leafy shoot a capacity for indeterminate growth. Through successive asymmetric divisions, the apical cell maintains itself and gives rise to merophytes, which divide to generate leaf initials and cells that produce stem tissues (Figure 2A). This cell-autonomous capacity to rotate cell division planes in 3D initiates the growth of upright leafy gametophores and underlies its spiral phyllotaxy (Figure 2B; Kamamoto et al., 2021; Véron et al., 2021). Similar to flowering plant meristems, leaf initial outgrowth and shoot apical cell function in mosses involve auxin and PIN-FORMED (PIN) mediated auxin transport but the precise mechanism of their action is unclear (Bennett et al., 2014). *SHORT-LEAF* (*SHLF*), a bryophyte specific tandem direct repeat gene, is likely involved in the underlying mechanism, as *SHLF* expression is associated with auxin accumulation in the gametophore and the capacity of the shoot apical cell to generate leaves (Mohanasundaram et al., 2021).

## Leaf Development

Leaf development in *P. patens* starts with the outgrowth of the leaf initial cell, which depends on auxin and cellulose biosynthesis (Goss et al., 2012; Bennett et al., 2014). The leaf initial cell maintains meristematic potential and cleaves daughter cells basipetally (Harrison et al., 2009). Similar to the shoot apical cell, the divisions of the leaf initial cell seem to be controlled cell-autonomously but instead of rotating spirally, subsequent divisions alternate in the same plane and are almost perpendicular to each other (Figures 2C,D; Harrison et al., 2009; Bascom et al., 2016). The orientation of the leaf initial cell division plane is likely controlled by microtubules as mutants lacking the cortical microtubule regulator TONNEAU1 develop thick multi-layered leaves (Spinner et al., 2010). Eventually, the leaf apical cell stops dividing and becomes the pointed tip of the leaf. Daughter cells derived from the leaf apical cell divide further, first near the leaf base (Figure 2E). The proliferative activity of these daughter cells decreases gradually so that cells near the tip divide less frequently and give rise to smaller segments of the leaf (Figures 2C,D; Harrison et al., 2009). Several rounds of longitudinal divisions within sectors derived from the leaf apical cell, especially in the outermost lateral portion of the leaf, lead to leaf broadening (Figure 2E). Additional transverse divisions also contribute to extending the daughter segments in the proximo-distal axis (Figure 2E; Harrison et al., 2009).

Quantitative analysis of the entire leaf growth at cellular resolution has not yet been performed in *P. patens*. However, the basipetal gradient of cell proliferation (Figure 2E; Harrison et al., 2009) and basipetal increase of cell sizes in mature leaves (Figure 2F; Barker and Ashton, 2013; Dennis et al., 2019) indicate that cells near the leaf tip are the earliest to cease growth. Cells at the tip differentiate first as they become insensitive to exogenous cytokinin, while cell proliferation in more proximal leaf regions is stimulated by this hormone (Barker and Ashton, 2013). Interestingly, basipetal gradients of growth, proliferation, and differentiation are key features of many flowering plant leaves and is controlled non-cell-autonomously by positional information (Avery, 1933; Andriankaja et al., 2012; Kuchen et al., 2012; Fox et al., 2018; Kierzkowski et al., 2019). Thus, apart from the cell-autonomous behavior of the leaf apical cell, positional cues also likely play a role in controlling moss leaf growth.

Auxin is a fundamental player in plant organogenesis where it regulates cell proliferation, elongation, and differentiation in a positional and context dependent manner (Vieten et al., 2007; Weijers et al., 2018). An essential common genetic toolbox involved in auxin biosynthesis, transport, and signaling is conserved between flowering plants and bryophytes (Poli et al., 2003; Kato et al., 2018; Thelander et al., 2018). Auxin could provide positional information during moss leaf development. Although the signal of the GH3:GUS reporter has not been observed in the wild type leaves of *P. patens*, exogenous auxin treatments disrupt leaf growth (Barker and Ashton, 2013; Bennett et al., 2014).

Auxin distribution within the developing leaf could be controlled by canonical auxin efflux carriers PIN-FORMED A and B (PpPINA and B) (Bennett et al., 2014; Viaene et al., 2014). The expression of *PpPINA* is displaced along the proximo-distal axis of the leaf during growth and correlates with developmental gradients (Figure 2G). This correlation is also evident at the subcellular level, where PpPINA protein is localized bipolarly on both apical and basal cell sides close to the leaf tip, while it is distributed more uniformly in cell membranes near the leaf base (Figure 2G; Viaene et al., 2014). Alternatively, auxin gradients in mosses might also be achieved by callose-controlled plasmodesmata-mediated diffusion (Coudert et al., 2015). Gametophores of the *shlf* mutant produce shorter leaves with a decreased cell number, similar to plants grown with a high auxin concentration or overexpressing *PpPINA*. Auxin activity gradients can be detected in leaves of the *shlf* mutant where GH3:GUS signal is present in very young leaves and at the tip of more developed leaves (Mohanasundaram et al., 2021). As *SHLF* might regulate plasmodesmata frequency, it could control leaf morphogenesis through the regulation of auxin gradients (Mohanasundaram et al., 2021).

Class III Homeodomain-Leucine Zipper (HD-ZIP III) transcription factors are key players during flowering plant development and are associated with auxin synthesis and transport (Prigge and Clark, 2006; Ariel et al., 2007; Turchi et al., 2015). There are five *HD-ZIP III* homologs in the *P. patens*, namely *PpC3HDZ1-5* (Yip et al., 2016). *PpC3HDZ* expression colocalizes with actively developing regions in adult leaves. Strikingly, *PpC3HDZ* knock-down induces pronounced, multicellular protrusions along leaf margins that resemble the distal portion of wild-type leaves. It indicates that in *PpC3HDZ* knock-down plants, daughter cells derived from the leaf apical cell, whose normal divisions produce characteristic leaf sectors (Figures 2C,D), could recapitulate the cell-autonomous behavior of the apical cell itself. Additionally, in *PpC3HDZ* knock-down leaves, cell number is reduced, suggesting that *HD-ZIP III* genes are important for the establishment and/or the maintenance of the proliferative activity of the daughter cells (Yip et al., 2016). *PpC3HDZ* expression domain mirrors PpPINA distribution, and both *PpC3HDZ* knock-down and *PpPINA/PpPINB* knock-out lines produce narrower leaves with a reduced cell number, indicating that *HD-ZIP III* function could be at least partially related to auxin-dependent positional information (Bennett et al., 2014; Viaene et al., 2014; Yip et al., 2016).

## Leaf Heteroblastic Development

Although all leaves of *P. patens* initiate from single cells, their morphology changes gradually up the gametophore axis (Figure 1A; Barker and Ashton, 2013; Dennis et al., 2019), a phenomenon called heteroblastic development (Zotz et al., 2011). As in *Arabidopsis*, juvenile leaves in *P. patens* are much smaller than adult leaves (Figures 1B,C; Courtice and Cove, 1983; Barker and Ashton, 2013). The width of the juvenile leaf is relatively constant except at the tapering tip, resulting in a roughly oblong shape (Figure 1C). By contrast, adult leaves have a lanceolate shape with a narrow base, a broader middle part, and a pointy tip (Figure 1B). The increase in size during heteroblastic development results from an increase in cell number but not cell size (Dennis et al., 2019). In flowering plants, auxin and cytokinin regulate cell proliferation and differentiation in an opposite manner during leaf development (Shani et al., 2010; Kierzkowski et al., 2019; Skalák et al., 2019). Exogenous treatments with auxin, or knock-out mutants in auxin efflux carriers *PpPINA* and *PpPINB*, cause a decrease in moss leaf size by reducing cell number (Bennett et al., 2014; Viaene et al., 2014). By contrast, cytokinin treatment increases cell proliferation and enhances adult leaf characteristics (Barker and Ashton, 2013). Altogether this indicates an important role of cytokinin-auxin cross-talk in moss leaf heteroblastic development.

The transition from juvenile to adult leaves in *P. patens* is also associated with morphological changes of the marginal cells that become long and narrow (Figure 1D) as well as the formation of a multilayered midrib (Figure 1E; Barker and Ashton, 2013; Dennis et al., 2019). Marginal cells tend to grow slightly outward at their distal end to form marginal serrations that usually appear on the apical half of the adult leaf (Barker and Ashton, 2013). The leaf margin in flowering plants has a distinct cell morphology and plays an important role during leaf development (Bilsborough et al., 2011; Nakata et al., 2012; Kalve et al., 2014; Alvarez et al., 2016; Hayakawa et al., 2016). Marginal cells in *P. patens* could play a role in controlling upper leaf size and shape by restricting or promoting growth around its circumference. The wrinkled leaves with undifferentiated marginal cells of *crinkly4* mutants likely result from mechanical conflicts in lamina cells (Demko et al., 2016). The leaf margin could also work in tandem with the midrib in the bigger leaves and ensure proper leaf flattening by preventing blade twisting. However, the role of marginal cells in moss leaf morphogenesis remains elusive.

## The Role of Midrib

Unlike vascular plants which conduct water through the xylem, mosses neither possess vessel elements or tracheids, nor have an interconnected network of veins. Instead, mosses mostly rely on external water conduction by capillary action to carry out water-dependent physiological functions (Proctor, 1979). However, the adult leaves conduct water through a multi-layered tissue called the midrib that is reminiscent of a vascular bundle and arises through a series of periclinal and anticlinal cell divisions (Ligrone et al., 2000; Sakakibara et al., 2003; Yip et al., 2016). These cell divisions give rise to different cell types within the leaf midrib, including thick-walled stereids and thin-walled, elongated hydroids (Figure 1E). Hydroids are the main water-conducting cells. They are initially alive, before undergoing programmed cell death, and have fully degenerate protoplasm upon maturation (Xu et al., 2014). Stereids also undergo programmed cell death but may mainly serve a supporting purpose. Therefore, hydroids resemble xylem vessel elements and tracheids, except that they lack a lignified secondary cell wall, while stereids resemble xylem fiber cells (Ligrone et al., 2000).

Several genes affecting midrib formation have been identified and characterized. For instance, among the seven moss genes that encode *VND/NST/SND* family transcription factors, six of them (*PpVNS1-7*, except *PpVNS3*) are expressed in the central region of newly formed leaves or in developing midribs. The triple mutant (*ppvns1,6,7*) forms morphologically normal midribs. However, stereid programmed cell death and hydroid proliferation are disrupted, and the water conducting capacity is greatly compromised (Xu et al., 2014). Interestingly, *VNS* orthologs are critical for xylem vessel element formation in angiosperms, suggesting that mosses and vascular plants may at least partly use the same molecular mechanism in shaping water-conducting tissue. The leaves of midrib-defective mutants tend to curl around the middle axis under low-humidity conditions, indicating a water transport deficiency. Nevertheless, compromised midrib formation does not seem to have a detrimental impact on overall moss growth, at least in laboratory conditions.

HD-ZIP III transcription factors also control midrib establishment. When their function is suppressed, midrib formation is abnormal and leaf shape becomes distorted (Yip et al., 2016). Given that HD-ZIP III transcription factors ATHB8, ATHB15, and REVOLUTA are essential for procambial cell specification and vasculature development, the observation that HD-ZIP III proteins function in specifying moss water-conducting tissues further suggests that shared molecular mechanisms underpin conducting tissue development in convergent plant organs (Kang and Dengler, 2002; Ohashi-Ito et al., 2002; Green et al., 2005; Prigge et al., 2005; Donner et al., 2009) and therefore that these mechanisms may have evolved before the divergence between bryophytes and vascular plants. Nonetheless, further research on midrib development is needed to uncover the regulatory circuits underlying the morphological differences between juvenile and adult leaves.

## Perspectives

In this review, we summarized the current understanding of leaf development in the model species *P. patens*. Proper leaf morphogenesis in this moss seems to require coordination of cell-autonomous and non-cell-autonomous developmental processes that are controlled via cross-talks between molecular regulators that are bryophyte-specific or shared by land plants. It is, however, still difficult to understand how these genetic and hormonal inputs are translated into cellular growth and division patterns and how they are coordinated in space and time within the mechanically connected tissue constituting the *P. patens* leaf.

In recent years, new research approaches combining genetics, quantitative live-imaging, biomechanics, and computational modeling have massively advanced our understanding of organ development in plants (Hervieux et al., 2016; Solly et al., 2017; Fox et al., 2018; Hong et al., 2018; Kierzkowski et al., 2019; Sapala et al., 2019; Wolny et al., 2020; Whitewoods et al., 2020; Hernandez-Lagana et al., 2021; Vijayan et al., 2021). The next step will be to apply such a multidisciplinary approach to study moss leaf development.

In comparison with the complex anatomy of flowering plant leaves, the single-cell-layered structure of *P. patens* leaf provides a unique opportunity to quantify the development of the entire organ in 3D. Such an approach, should provide a comprehensive picture of moss leaf developmental dynamics at cellular resolution that otherwise would be difficult to apprehend. In contrast with flowering plants, gene targeting by homologous recombination is very efficient in *P. patens*, and together with CRISPR/Cas9-mediated gene editing, permits to bypass genetic redundancy and rapidly generate high-order mutants (Koshimizu et al., 2018). Combination of imaging and genetic approaches will, for example, help us to understand the precise role of molecular regulators in the control of cell-autonomous and non-cell-autonomous behaviors during growth and their role in juvenile to adult leaf transition.

Single-cell-layered leaves in *P. patens* will also be a huge asset to dissect the role of biomechanical signals regulating plant organogenesis. Largely eliminating complex interactions that occur between different tissue layers of developing organs, as in flowering plants, this moss model system should provide a better understanding of the mechanical interactions between individual cells and their role in the coordination of organ growth. As *P. patens* leaves are easy to manipulate and have relatively big cells, they should also enable precise measurements of cell mechanical properties using modern micro-indentation devices (Routier-Kierzkowska et al., 2012; Robinson et al., 2017; Majda et al., 2019) or turgor pressure manipulations (Kierzkowski et al., 2012; Sapala and Smith, 2020). All these experimental inputs, in combination with geometrically accurate templates extracted from confocal images, will enable the creation of biologically realistic simulations of the entire moss leaf. Such models will not only provide a comprehensive picture of *P. patens* leaf development, but also advance our general understanding of the mechanism governing plant organogenesis.

## Author Contributions

WL, YW, YC, and DK wrote and edited the article. All authors contributed to the article and approved the submitted version.

## Conflict of Interest

The authors declare that the research was conducted in the absence of any commercial or financial relationships that could be construed as a potential conflict of interest.

## Publisher’s Note

All claims expressed in this article are solely those of the authors and do not necessarily represent those of their affiliated organizations, or those of the publisher, the editors and the reviewers. Any product that may be evaluated in this article, or claim that may be made by its manufacturer, is not guaranteed or endorsed by the publisher.
